# Tract-specific differences in white matter microstructure between young adult *APOE* ε4 carriers and non-carriers: A replication and extension study

**DOI:** 10.1016/j.ynirp.2022.100126

**Published:** 2022-12

**Authors:** Rikki Lissaman, Thomas M. Lancaster, Greg D. Parker, Kim S. Graham, Andrew D. Lawrence, Carl J. Hodgetts

**Affiliations:** a Cardiff University Brain Research Imaging Centre (CUBRIC), School of Psychology, Cardiff University, Cardiff, Wales, United Kingdom; b Douglas Research Centre, Montreal, Quebec, Canada; c Department of Psychiatry, McGill University, Montreal, Quebec, Canada; d School of Psychology, University of Bath, Bath, England, United Kingdom; e Department of Psychology, University of Edinburgh, Edinburgh, Scotland, United Kingdom; f Department of Psychology, Royal Holloway, University of London, Egham, England, United Kingdom

**Keywords:** *APOE*, Alzheimer's disease, Parahippocampal cingulum bundle, Inferior longitudinal fasciculus, Diffusion MRI, Structural connectivity

## Abstract

The parahippocampal cingulum bundle (PHCB) interconnects regions known to be vulnerable to early Alzheimer's disease (AD) pathology, including posteromedial cortex and medial temporal lobe. While AD-related pathology has been robustly associated with alterations in PHCB microstructure, specifically lower fractional anisotropy (FA) and higher mean diffusivity (MD), emerging evidence indicates that the reverse pattern is evident in younger adults at increased risk of AD. In one such study, Hodgetts et al. (2019) reported that healthy young adult carriers of the apolipoprotein-E (*APOE*) ε4 allele – the strongest common genetic risk factor for AD – showed higher FA and lower MD in the PHCB but not the inferior longitudinal fasciculus (ILF). These results are consistent with proposals claiming that heightened neural activity and intrinsic connectivity play a significant role in increasing posteromedial cortex vulnerability to amyloid-β and tau spread beyond the medial temporal lobe. Given the implications for understanding AD risk, here we sought to replicate Hodgetts et al.‘s finding in a larger sample (*N* = 128; 40 *APOE* ε4 carriers, 88 *APOE* ε4 non-carriers) of young adults (age range = 19–33). Extending this work, we also conducted an exploratory analysis using a more advanced measure of white matter microstructure: hindrance modulated orientational anisotropy (HMOA). Contrary to the original study, we did not observe higher FA or lower MD in the PHCB of *APOE* ε4 carriers relative to non-carriers. Bayes factors (BFs) further revealed moderate-to-strong evidence in support of these null findings. In addition, we observed no *APOE* ε4-related differences in PHCB HMOA. Our findings indicate that young adult *APOE* ε4 carriers and non-carriers do not differ in PHCB microstructure, casting some doubt on the notion that early-life variation in PHCB tract microstructure might enhance vulnerability to amyloid-β accumulation and/or tau spread.

## Highlights

• Posteromedial network especially vulnerable to early Alzheimer's disease (AD).
• Altered posteromedial network connectivity linked to AD genetic risk (*APOE* ε4).
• Parahippocampal cingulum bundle (PHCB) is a key tract within posteromedial network.
• Prior dMRI study found altered PHCB microstructure in young adult *APOE* ε4 carriers.
• Found evidence against altered PHCB microstructure in young adult *APOE* ε4 carriers.

## Graphical abstract

**Figure:**
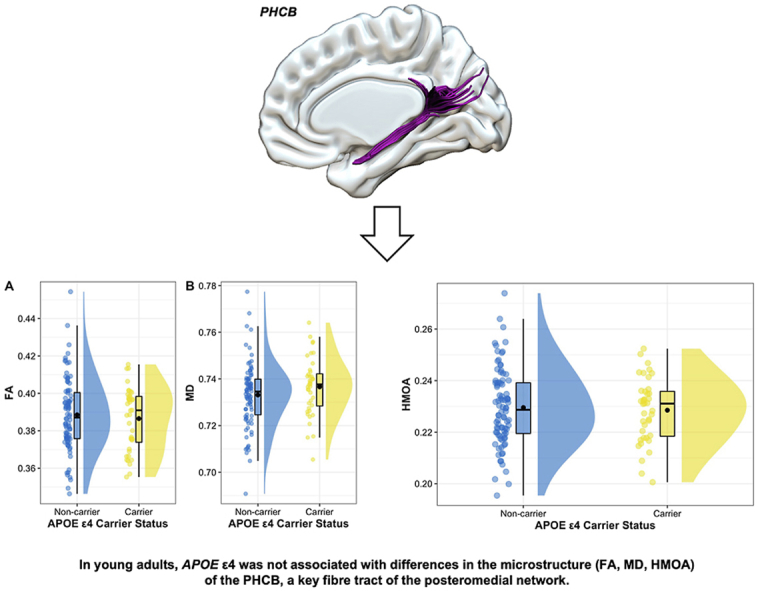

## 1 Introduction

Alzheimer's disease (AD) is a chronic, progressive disease and the most common cause of dementia (Scheltens et al., 2021). The hallmark pathological features of AD are the presence of extracellular amyloid-β-containing plaques and intracellular tau-containing neurofibrillary tangles (DeTure and Dickson, 2019; Trejo-Lopez et al., 2022). Although controversial (Frisoni et al., 2022; Herrup, 2015), the dominant hypothesis in the field – the amyloid cascade hypothesis – holds that the accumulation of amyloid-β peptide is the critical factor in AD pathogenesis (Selkoe and Hardy, 2016). Amyloid-β accumulation follows a relatively distinct spatiotemporal pattern in the ageing brain, beginning preferentially in posteromedial regions, including retrosplenial/posterior cingulate cortices and precuneus (Mattsson et al., 2019; Palmqvist et al., 2017; Villeneuve et al., 2015). Collectively, these regions are sometimes referred to as posteromedial cortex (Parvizi et al., 2006). The vulnerability of posteromedial cortex to AD pathology has been linked to its hub-like properties (Jagust, 2018; Yu et al., 2021), in particular its high-levels of baseline metabolic/neural activity and high intrinsic/extrinsic connectivity (Bero et al., 2012; Buckner et al., 2009; de Haan et al., 2012; Jagust and Mormino, 2011; Jones et al., 2016; Myers et al., 2014). Notably, posteromedial cortex is densely connected with several medial temporal lobe structures (e.g., parahippocampal cortex and hippocampus) and thus forms a “posterior medial” or “extended navigation” network (Murray et al., 2016; Ranganath and Ritchey, 2012), a subsystem of the default network (Raichle, 2015; Ritchey and Cooper, 2020). This network is implicated in several inter-related cognitive functions that are impaired early in AD, such as episodic memory (Rajah et al., 2017), perceptual scene discrimination (Lee et al., 2006), and spatial navigation (Coughlan et al., 2018). Given this, there is a pressing need to identify biomarkers that capture the connectivity of this AD-vulnerable brain network. In this context, the parahippocampal cingulum bundle (PHCB) – a prominent long-range white matter tract linking posteromedial cortex with the medial temporal lobe (Bubb et al., 2018; Heilbronner and Haber, 2014; Jitsuishi and Yamaguchi, 2021) – represents a strong candidate for understanding and characterising structural connectivity alterations associated with AD.

Increasing evidence indicates that PHCB connectivity is altered in AD. Using diffusion magnetic resonance imaging (dMRI) – a non-invasive method that examines the random, microscopic movement of water molecules – it is possible to delineate the major white matter tracts of the brain and evaluate their microstructural properties in vivo (Assaf et al., 2019; Jbabdi and Behrens, 2013). In most AD-relevant dMRI studies, white matter microstructure is assessed via measures derived from the diffusion tensor model (Basser, 1997), notably fractional anisotropy (FA) and mean diffusivity (MD) (Harrison et al., 2020). Although multiple biological factors can influence these measures (Jones et al., 2013a), they are often used as indices of connectivity (Yeh et al., 2016; Yeh et al., 2021), with lower FA and higher MD values interpreted as *lower* connectivity. Studies comparing AD patients to cognitively normal older adults using dMRI have reliably observed both lower FA and higher MD in the cingulum bundle and the PHCB in particular (Acosta-Cabronero et al., 2010; Bozzali et al., 2012; Choo et al., 2010; Kantarci et al., 2017; Salat et al., 2010; Villain et al., 2008; Zhang et al., 2007). Such changes are functionally relevant, being linked both to disrupted functional connectivity of the posteromedial network (Zhou et al., 2022) and impaired episodic memory (Berron et al., 2020; Fellgiebel et al., 2008). In addition, longitudinal changes in PHCB microstructure – reduced FA, increased MD – have been reported among AD patients but not cognitively normal older adults (Mayo et al., 2017). Indeed, it has recently been suggested that PHCB FA constitutes a highly effective biomarker for differentiating between typical ageing and AD (Dalboni da Rocha et al., 2020).

Studies of amnestic mild cognitive impairment (aMCI) – a transitional stage between typical ageing and AD (Albert et al., 2011) – further highlight that PHCB alterations precede the onset of AD dementia. In one region-of-interest (ROI) meta-analysis, for example, Yu et al. (2017) identified robust alterations in PHCB microstructure (lower FA, higher MD) among individuals with aMCI. This is consistent with the notion that cingulum bundle alterations predict cognitive decline in aMCI, and may even predict conversion to AD (Gozdas et al., 2020). Studies combining positron emission tomography and dMRI have also allowed PHCB changes to be linked directly to AD pathology. For example, amyloid-β burden has been associated with longitudinal changes in white matter microstructure that are consistent with patterns observed in aMCI and AD (Rieckmann et al., 2016; Song et al., 2018; Vipin et al., 2019). In particular, high levels of cortical amyloid-β burden at baseline have been associated with accelerated decline in PHCB FA and a trend-level increase in PHCB MD (Rieckmann et al., 2016). In keeping with this tract-specific finding, one recent cross-sectional study reported that lower FA and higher MD in the PHCB was associated with greater cortical amyloid-β and entorhinal tau burden, especially in those with high levels of pre-existing pathology (Pichet Binette et al., 2021; although see Vlegels et al., 2022). It thus appears that PHCB microstructure is detrimentally impacted over the course of AD, including stages prior to the onset of dementia symptoms (see also Alm et al., 2022).

Emerging research indicates, however, that asymptomatic individuals exhibit alterations in white matter microstructure that run counter to the characteristic AD pattern. Illustrating this point, several cross-sectional studies have observed higher FA and lower MD in early-stage amyloid-β pathology, a pattern that is reversed as pathology further accrues (Collij et al., 2021; Dong et al., 2020; Wolf et al., 2015). These findings point to a potential biphasic relationship between amyloid-β and white matter microstructure, with a period of higher FA/lower MD preceding the pattern commonly observed in patients with aMCI and AD. Despite this, longitudinal data are currently lacking and, therefore, questions remain about the nature of the relationship between amyloid-β and white matter microstructure.

One set of proposals states that heightened posteromedial intrinsic connectivity – at least partly underpinned by structural connectivity (Suárez et al., 2020) – may actually predispose individuals to amyloid-β deposition in later life (Bero et al., 2012; Buckner et al., 2009; de Haan et al., 2012; Jagust and Mormino, 2011; Myers et al., 2014). Support for this proposal may be found in studies of young adult carriers of the apolipoprotein-E (*APOE*) ε4 allele, the strongest common genetic risk factor for AD (Belloy et al., 2019). Although not all individuals possessing the *APOE* ε4 allele go on to develop AD, the probability that a randomly selected individual with the ε4/ε4 or ε3/ε4 genotype will develop AD by age 85 is estimated to be 51–60% and 23–30%, respectively (Genin et al., 2011). Accordingly, a recent probabilistic model of AD proposed that *APOE* ε4 carrier status should be considered a major effect modifier, increasing the penetrance of the amyloid-β cascade (Frisoni et al., 2022). Indeed, *APOE* ε4 is associated with a younger age of onset and faster rate of posteromedial amyloid-β accumulation (Burnham et al., 2020; Mishra et al., 2018). In line with the notion that this amyloid-β accumulation may be related to earlier connectivity changes, a study applying graph theoretical analysis to dMRI data observed that age was negatively associated with local interconnectivity in posteromedial regions, but only among *APOE* ε4 carriers (Brown et al., 2011). Higher levels of local interconnectivity in their sample's younger participants drove this finding (age range = 43–78 years), such that there was a putative *APOE* ε4-related increase in connectivity early in life that was subsequently followed by a sharper decline later in life (Brown et al., 2011; see also Ma et al., 2017). Felsky and Voineskos (2013) further reported higher cingulum bundle FA in younger *APOE* ε4 carriers compared to younger non-carriers (∼20–40 years), but lower cingulum bundle FA in middle-aged and older *APOE* ε4 carriers compared to non-carriers (>50 years; see also Heise et al., 2014). Given that younger adults are unlikely to possess significant amyloid-β burden (Jansen et al., 2015), these findings suggest that early-life structural variation may – along with other risk and/or protective factors (Silva et al., 2019) – increase vulnerability to amyloid-β in later life.

Consistent with this, Hodgetts et al. (2019) observed higher FA and lower MD among young adult *APOE* ε4 carriers (mean age = 19.7 years) relative to non-carriers (mean age = 19.7 years) in the PHCB but not the inferior longitudinal fasciculus (ILF), a tract that connects the occipital lobe to the ventro-anterior temporal lobe (Herbet et al., 2018). Hodgetts et al. also found that PHCB microstructure was correlated with posteromedial cortex activity during perceptual scene discrimination, a task that has previously been shown to elicit heightened posteromedial cortex activity in young *APOE* ε4 carriers (Shine et al., 2015) and is sensitive to AD (Lee et al., 2006). Given the proposal that heightened posteromedial neural activity and intrinsic connectivity increase hub-like vulnerability to amyloid-β (Bero et al., 2012; Buckner et al., 2009; de Haan et al., 2012; Jagust and Mormino, 2011; Myers et al., 2014), as well as the tight coupling between posteromedial network structural and intrinsic functional connectivity (Damoiseaux and Greicius, 2009), it is plausible that such early-life differences in PHCB microstructure may partly explain why *APOE* ε4 is associated with increased risk of earlier and faster posteromedial amyloid-β accumulation (Burnham et al., 2020; Mishra et al., 2018). Furthermore, as the spread of tau has been linked to heightened functional connectivity between posteromedial cortex and the medial temporal lobe (Guzmán-Vélez et al., 2022; Ziontz et al., 2021) – presumably mediated by the PHCB (Jacobs et al., 2018) – it is possible that early-life increases in structural connectivity are also related to enhanced risk of elevated tau in *APOE* ε4 carriers (Therriault et al., 2020).

In view of the potential implications, we sought to replicate Hodgetts et al.’s (2019) finding that healthy young adult *APOE* ε4 carriers demonstrate higher FA and lower MD than non-carriers in the PHCB but not the ILF. We analysed data from an independent data set of young adults, with a total sample over four times larger than the original study. This replication attempt thus constitutes an important test of the notion that increased PHCB structural connectivity, as indexed by higher FA and lower MD, is evident in young adult *APOE* ε4 carriers, potentially increasing network vulnerability to both amyloid-β accumulation and tau spread in later life. We also report an additional exploratory analysis that seeks to extend this work by incorporating a more advanced measure of microstructure: hindrance modulated orientational anisotropy (HMOA; Dell’Acqua et al., 2013). Unlike measures derived from the diffusion tensor model, HMOA is able to account for the presence of crossing fibres and is therefore considered a tract-specific measure of white matter microstructure (Dell’Acqua and Tournier, 2019). Using simulations, HMOA has further been shown to be more sensitive to alterations in anisotropy than either FA or MD (Dell’Acqua et al., 2013). Additional support for this assertion can be found in studies reporting that HMOA is able to detect white matter variation linked to verbal memory (Christiansen et al., 2016) and ageing (Rojkova et al., 2016) not detectable with standard diffusion tensor-derived measures. As such, we investigated whether *APOE* ε4 is associated with differences in PHCB and ILF HMOA, complementing the primary (replication) analyses.

## 2 Method

### 2.1 Participants

Participant data were acquired from a repository at the Cardiff University Brain Research Imaging Centre. Portions of this data have been published elsewhere (Foley et al., 2017; Koelewijn et al., 2019). Participants were healthy adults, who were screened via interview or questionnaire for the presence of neuropsychiatric disorders. All were right-handed, had normal or corrected-to-normal vision, and provided informed consent for their data to be used in imaging genetics analyses. All procedures were originally reviewed and approved by the Cardiff University School of Psychology Research Ethics Committee. For the current study, participants were only included if they completed the requisite MRI scans, had *APOE* genotype information available, and were aged 35 years or under (*N* = 148). This age cut-off mirrors that used by other neuroimaging studies examining the effect of *APOE* genotype on brain structure and/or function in young adults (for examples, see Filippini et al., 2009, 2011; Stening et al., 2017). After additional exclusions were applied – described below (see also Supplementary Fig. 1) – the final sample comprised 128 participants (86 females, 42 males) aged between 19 and 33 years (*M* = 23.8, *SD* = 3.6).

Consistent with Hodgetts et al. (2019), the final sample was split into carrier and non-carrier groups based on the presence/absence of the *APOE* ε4 allele. Participants carrying both risk-enhancing (ε4) and risk-reducing (ε2) *APOE* alleles were included as part of the carrier group, as the ε2ε4 genotype is associated with higher levels of AD pathology and risk (Goldberg et al., 2020; Jansen et al., 2015; Reiman et al., 2020). Although *APOE* is often directly genotyped, as in Hodgetts et al.‘s study, here it was inferred from imputed (1000G phase 1, version 3) genome-wide genetic data (for more detail, see Foley et al., 2017). Previous research has demonstrated that it is possible to accurately infer *APOE* genotypes using this method (Lupton et al., 2018; Oldmeadow et al., 2014; Radmanesh et al., 2014). Overall, the current sample included 40 *APOE* ε4 carriers (4 ε2/ε4, 33 ε3/ε4, 3 ε4/ε4) and 88 *APOE* ε4 non-carriers (4 ε2/ε2, 14 ε2/ε3, 70 ε3/ε3). An effect size sensitivity analysis calculated using the *pwr* package (version 1.3–0; Champely et al., 2020) in R (version 4.1.3; R Core Team, 2022) revealed that the smallest effect size detectable at 80% power was Cohen's *d*_*s*_ = 0.575 (1-β = 0.80, Bonferroni-corrected α = 0.016, directional hypothesis). By comparison, even without correcting the α level for multiple comparisons, the smallest effect size detectable at 80% power in Hodgetts et al.‘s study was Cohen's *d*_*s*_ = 0.931 (1-β = 0.80, α = 0.05, directional hypothesis). Basic sample characteristics for this study and for Hodgetts et al.‘s study are highlighted in Table 1.

**Table 1:** Basic sample characteristics in the current study and in Hodgetts et al. (2019).

	Current Study		Hodgetts et al. (2019)	
	*APOE* ε4+ (*n* = 40)	*APOE* ε4-(*n* = 88)	*APOE* ε4+ (*n* = 15)	*APOE* ε4-(*n* = 15)

Age (years)	23.9 ± 3.3	23.7 ± 3.7	19.7 ± 0.84	19.7 ± 0.89
Sex (Males/Females)^a^	12/28	30/58	1/14	1/14
*APOE* genotype	4 ε2/ε4,	4 ε2/ε2,	1 ε2/ε4,	0 ε2/ε2,
	33 ε3/ε4,	14 ε2/ε3,	14 ε3/ε2,	5 ε2/ε3,
	3 ε4/ε4	70 ε3/ε3	0 ε4/ε4	10 ε3/ε3

*Note*. For age, values represent the mean and standard deviation. For *APOE* genotype and sex, values represent the number of participants. Abbreviations: *APOE* ε4+ = *APOE* ε4 carrier, *APOE* ε4- = *APOE* ε4 non-carrier.

a Although sex was self-reported in the current study, it was checked against chromosomal sex as part of genetic quality control procedures (Foley et al., 2017).

### 2.2 MRI scan parameters

As in Hodgetts et al. (2019), scanning was conducted on a GE SIGNA HDx 3T MRI system (General Electric Healthcare, Milwaukee, WI) with an eight-channel receive-only head coil. Whole-brain high angular resolution diffusion imaging data (Tuch et al., 2002) were acquired using a diffusion-weighted single-shot echo-planar imaging sequence (TE = 89 ms; voxel dimensions = 2.4 × 2.4 × 2.4 mm; FOV = 230 mm × 230 mm; acquisition matrix = 96 × 96; 60 slices aligned AC/PC with 2.4 mm thickness and no gap). Gradients were applied along 30 isotropic directions (Jones et al., 1999) with b = 1200 s/mm^2^. Three non-diffusion-weighted images were acquired with b = 0 s/mm^2^. Acquisitions were cardiac-gated using a peripheral pulse oximeter. T1-weighted anatomical images were acquired using a three-dimensional fast spoiled gradient-echo sequence (TR/TE = 7.8/3s; voxel dimensions = 1 mm isotropic; FOV ranging from 256 × 256 × 168 mm to 256 × 256 × 180 mm; acquisition matrix ranging from 256 × 256 x 168 to 256 × 256 x 180; flip angle = 20°). These sequences were similar to those used by Hodgetts et al. (2019), with only subtle differences between the two studies (outlined in Supplementary Table 1).

### 2.3 dMRI

#### 2.3.1 Pre-processing

The dMRI data were corrected for motion- and eddy current-induced distortions in ExploreDTI (version 4.8.6; Leemans et al., 2009), with an appropriate reorientation of the b-matrix (Leemans and Jones, 2009). Images were registered to down-sampled T1-weighted images (1.5 mm isotropic resolution) to correct for susceptibility deformations (Irfanoglu et al., 2012). Data were visually checked as part of quality assurance procedures, leading to the removal of two participants from the analysis due to poor quality data. Consistent with Hodgetts et al. (2019), the two-compartment free-water elimination procedure was implemented using in-house MATLAB code (version R2015a; MathWorks, Inc., 2015) to correct for voxel-wise partial volume artefacts (Pasternak et al., 2009). This procedure has been shown to improve tract delineation, as well as the sensitivity and specificity of measures traditionally derived from the diffusion tensor (Pasternak et al., 2009). Free-water corrected FA and MD maps were then used in further analyses. FA represents the degree to which diffusion is constrained in a particular direction, ranging from 0 (isotropic diffusion) to 1 (anisotropic diffusion). By contrast, MD (10^−3^mm^2^s^−1^) represents the average diffusivity rate.

#### 2.3.2 Tractography

The RESDORE algorithm was used to identify outliers in the diffusion data (Parker, 2014), and then tractography was conducted in ExploreDTI using the modified damped Richardson-Lucy spherical deconvolution algorithm (Dell’Acqua et al., 2010). Spherical deconvolution approaches enable multiple peaks to be extracted in the white matter fibre orientation density function (fODF) within a given voxel. This allows complex fibre arrangements, such as crossing/kissing fibres, to be modelled more accurately (Dell’Acqua and Tournier, 2019). The current study and the original study by Hodgetts et al. (2019) both used spherical deconvolution approaches, although the latter used the constrained spherical deconvolution algorithm (Jeurissen et al., 2011). While this might lead to subtle differences between the two studies, the modified damped Richardson-Lucy deconvolution algorithm was selected here because it is considered less sensitive to miscalibration (Parker et al., 2013). To minimise any further discrepancies between the studies, tracts were reconstructed using the same parameters used by Hodgetts et al. (fODF amplitude threshold = 0.1; step size = 0.5 mm; angle threshold = 60°).

**Fig. 1:**
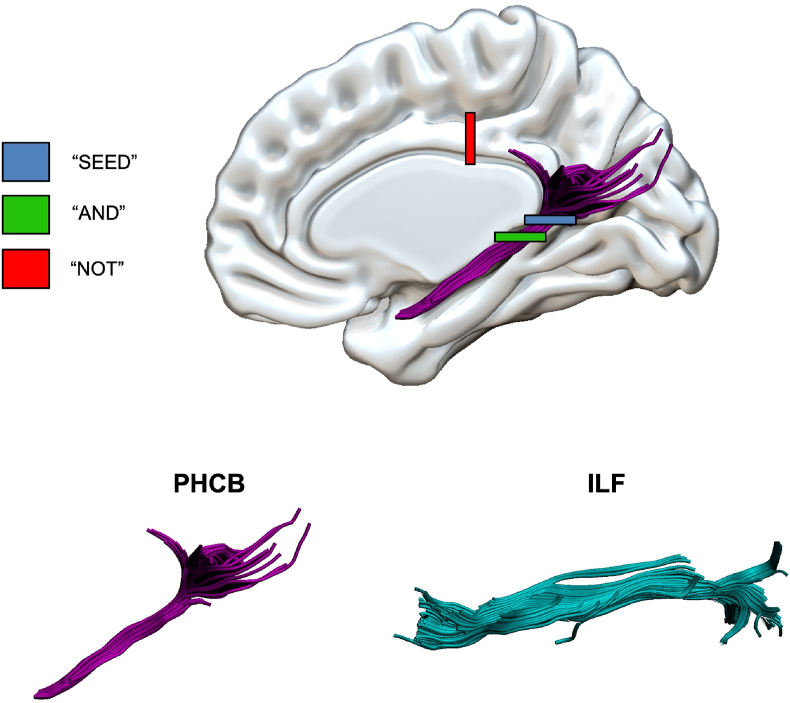
*Manual Reconstructions of the PHCB and ILF* *Note*. “SEED”, “AND”, and “NOT” ROIs used to manually reconstruct the PHCB are highlighted. Example tract reconstructions are also shown for both the PHCB and ILF. The resulting tracts were used to train the semi-automated tractography software (Parker et al., 2012) and produce tracts for the entire sample. Abbreviations: ILF = inferior longitudinal fasciculus, PHCB = parahippocampal cingulum bundle, ROI = region of interest.

In-house semi-automated tractography software (Parker et al., 2012) was used to generate three-dimensional reconstructions of the PHCB and ILF in both hemispheres. The software was trained on manual reconstructions generated by author R.L. using a waypoint ROI approach in ExploreDTI, where “SEED”, “AND”, and “NOT” ROIs were used to isolate tract-specific streamlines (Fig. 1). ROIs were placed in the same regions as described by Hodgetts et al. (2019). Placement was therefore guided by established protocols for the PHCB (Jones et al., 2013b) and the ILF (Wakana et al., 2007), respectively. All reconstructions generated by the semi-automated software were visually inspected by authors R.L. and C.J.H. and, where required, manually edited post hoc to remove erroneous, anatomically implausible fibres. Participants for whom the PHCB and ILF could not be reconstructed in both hemispheres were removed from analysis (*n* = 18). Thereafter, measures of microstructure were obtained and averaged across tracts. Although the semi-automated approach used here differs from that used by Hodgetts et al., it is considerably less time-consuming and arguably less prone to user error, especially when working with larger samples (*N* > 100). It is for this reason that studies recruiting samples of equivalent size have likewise adopted a semi-automated approach (for relevant examples, see Foley et al., 2017; Metzler-Baddeley et al., 2019). Nevertheless, during visual inspection, author C.J.H. further confirmed that tract reconstruction produced qualitatively similar outputs to those obtained in the original, to-be-replicated study.

#### 2.3.3 Tract-based spatial statistics (TBSS)

Complementary voxel-wise statistical analysis of the FA and MD data was conducted using TBSS (Smith et al., 2006). Each participant's free-water corrected FA and MD maps were first aligned in standard MNI space using nonlinear registration (Andersson et al., 2007a, 2007b). Next, the mean FA image was created and subsequently thinned (threshold = 0.2) to generate the mean FA skeleton, which represents the centre of all tracts common to the group. Each participant's aligned FA and MD data were then projected onto the skeleton and the resulting data carried forward for voxel-wise cross-subject analysis. These analyses were performed using *randomise* (Winkler et al., 2014), a permutation-based inference tool. For both FA and MD, a general linear model contrasting *APOE* ε4 carriers and non-carriers (FA: carrier > non-carrier; MD: carrier < non-carrier) was applied (*n* permutations = 1000). Mirroring Hodgetts et al.’s (2019) example, analyses were first restricted to the PHCB using an ROI mask [labelled “cingulum (hippo-campus)”] from the John Hopkins University ICBM-DTI-81 white-matter tractography atlas. An exploratory whole-brain analysis was then conducted. Statistically significant clusters were extracted from both analyses using threshold-free cluster enhancement with a corrected α level of 0.05 (Smith and Nichols, 2009).

### 2.4 Statistical analyses

Except for TBSS, all statistical analyses were conducted using R. In addition to common frequentist null hypothesis significance tests, Bayes factors (BFs) were calculated. BFs quantify the degree to which the observed data favours predictions made by two models, in this case the null hypothesis and the alternative hypothesis. Consequently, BF analyses can provide evidence in support of the null (Dienes, 2014). In accordance with the evidence categories outlined by Lee and Wagenmakers (2013), a BF_+0_ (BF_10_ for two-sided tests) greater than 3 was considered to represent at least moderate evidence for the alternative hypothesis, whereas a BF_+0_ less than 0.33 was considered to represent at least moderate evidence for the null hypothesis.

#### 2.4.1 Primary (replication) analyses

To test whether *APOE* ε4 carriers showed higher FA and lower MD in the PHCB but not the ILF, one-sided Welch's *t*-tests were conducted. As in Hodgetts et al. (2019), all tests were repeated, once with male participants removed and once with ε2 carriers removed. These additional tests – performed independently of each other – were originally conducted based on evidence that *APOE* ε4 may have a stronger effect on AD biomarkers in females than males (Riedel et al., 2016; Subramaniapillai et al., 2021; Wang et al., 2021), whereas *APOE* ε2 may have a protective effect on AD biomarkers (Suri et al., 2013; Reiman et al., 2020). Given that the same hypothesis was tested three times, a Bonferroni correction was applied to control the family-wise error rate (0.05/3 = 0.016). Two BFs were also calculated: a default JZS BF and a replication BF. The default JZS BF, which uses a default prior distribution and was computed using the *BayesFactor* package (version 0.9.12–4.4; Morey et al., 2022), examines whether an effect is present or absent in the data collected in the replication study regardless of the original effect. Here, one-sided (directional) default JZS BFs were calculated. The replication BF, by contrast, uses the posterior distribution of the original study as the prior distribution in the replication study, examining whether the original effect is present or absent in the data collected in the replication study. This BF was computed using previously published R code (Verhagen and Wagenmakers, 2014).

#### 2.4.2 Secondary (extension) analyses

It remains to be seen whether *APOE* ε4-related differences in PHCB microstructure are better captured by measures other than FA and MD, which are detrimentally affected by the presence of crossing fibres (Jones et al., 2013a). One such measure is HMOA (Dell’Acqua et al., 2013), which is defined as the absolute amplitude of each fODF lobe normalised to a reference amplitude (the highest possible diffusion value detectable in biological tissue). Normalisation ensures that HMOA has a range of zero to one, where zero reflects the absence of a fibre and one reflects maximum diffusivity (Dell’Acqua et al., 2013).

Given the lack of a directional hypothesis relating to HMOA, two-sided Welch's *t*-tests and two-sided default JZS BFs were used to identify any differences between *APOE* ε4 carriers and non-carriers. In keeping with the primary (replication) analyses described above, these tests were repeated with males removed and then with ε2 carriers removed. These analytical steps were performed independently. A Bonferroni correction aimed at controlling the family-wise error rate was again applied to the nominal α level (0.05/3 = 0.016).

### 2.5 Data and code availability

R code used to analyse and visualise data in the current study is made publicly available via the Open Science Framework (https://osf.io/f6jp3/). Due to the sensitive nature of the data, the original ethics do not allow for the public archiving of study data (for more information, see Koelewijn et al., 2019). Access to pseudo-anonymised data may be granted, however, after the signing and approval of suitable data-transfer agreements. Readers seeking access through this mechanism should contact Professor Krish D. Singh at the Cardiff University Brain Research Imaging Centre (singhkd@cardiff.ac.uk).

## 3 Results

### 3.1 Primary (replication) analyses

#### 3.1.1 Effect of *APOE* ε4 on PHCB FA and MD

**Fig. 2:**
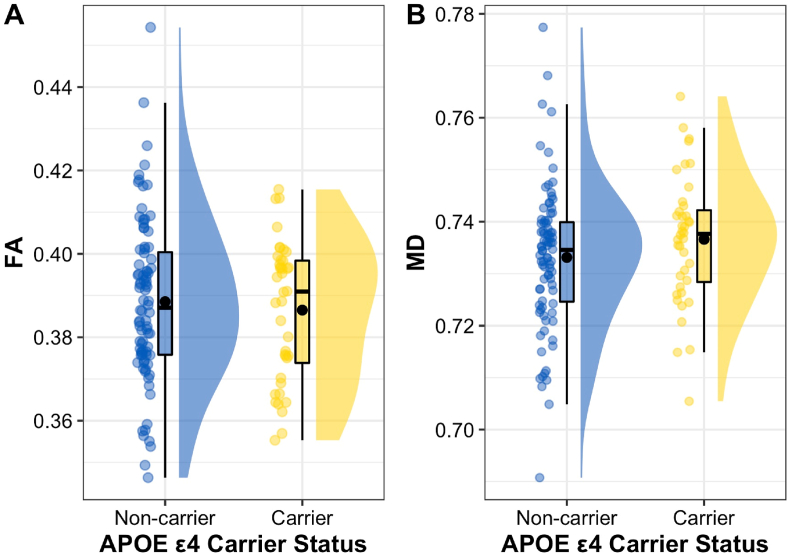
*Differences in PHCB FA and MD Between APOE ε4 Carriers and Non-Carriers* *Note*. Differences in (A) PHCB FA (range = 0–1) and (B) MD (10^−3^mm^2^s^−1^) between *APOE* ε4 carriers and non-carriers are shown. Individual data points, each representing a single participant, are shown alongside boxplots and density plots (“raincloud plots”; Allen et al., 2021). A small amount of jitter has been added to each data point for clarity. To facilitate interpretation, the mean value (black circle) and median value (a black line) for each group are both shown. Abbreviations: FA = fractional anisotropy, MD = mean diffusivity.

FA values for the PHCB – separated by *APOE* ε4 carrier status – are shown in Fig. 2A. Contrary to our initial hypothesis, PHCB FA was not significantly higher for *APOE* ε4 carriers than non-carriers (*t*(87.559) = −0.606, *p* = .727, Cohen's *d*_*s*_ = −0.112). Supporting this, BF analysis produced moderate evidence in favour of the null (default JZS BF_+0_ = 0.138, replication BF_10_ = 0.141). Removing males from the analysis did not alter the results in any meaningful way (*t*(57.685) = 0.045, *p* = .482, Cohen's *d*_*s*_ = 0.01, default JZS BF_+0_ = 0.246, replication BF_10_ = 0.168), nor did removing ε2 carriers (*t*(84.459) = −0.923, *p* = .821, Cohen's *d*_*s*_ = −0.183, default JZS BF_+0_ = 0.125, replication BF_10_ = 0.271).

MD values for the PHCB – separated by *APOE* ε4 carrier status – are shown in Fig. 2B. Again, contrary to prior expectations, PHCB MD was not significantly lower for *APOE* ε4 carriers than non-carriers (*t*(83.625) = 1.429, *p* = .922, Cohen's *d*_*s*_ = 0.267). Here, BF analysis revealed strong evidence in favour of the null (default JZS BF_+0_ = 0.092, replication BF_10_ = 0.057). As with FA, the results for MD did not change substantively after removing males (*t*(59.729) = 1.515, *p* = .933, Cohen's *d*_*s*_ = 0.341, default JZS BF_+0_ = 0.106, replication BF_10_ = 0.054) or after removing ε2 carriers (*t*(79.581) = 1.328, *p* = .906, Cohen's *d*_*s*_ = 0.267, default JZS BF_+0_ = 0.103, replication BF_10_ = 0.1).

#### 3.1.2 Effect of *APOE* ε4 on ILF FA and MD

The same analysis was conducted on ILF FA and MD. Analysis revealed that ILF FA was not significantly higher for *APOE* ε4 carriers than non-carriers (*t*(86.143) = −0.864, *p* = .805, Cohen's *d*_*s*_ = −0.16). BF analysis provided moderate-to-strong evidence favouring the absence of an effect (default JZS BF_+0_ = 0.12), as well as anecdotal-to-moderate evidence favouring the absence of the effect reported by Hodgetts et al. (replication BF_10_ = 0.309). This slight discrepancy between BFs is likely because the original to-be-replicated effect was also small and did not reach the threshold for statistical significance, meaning that the informed prior used was already more “sceptical” than the default prior. Results remained largely unchanged when males were removed (*t*(49.129) = −0.069, *p* = .527, Cohen's *d*_*s*_ = −0.016, default JZS BF_+0_ = 0.226, replication BF_10_ = 0.308) and when ε2 carriers were removed (*t*(79.5) = −0.893, *p* = .813, Cohen's *d*_*s*_ = −0.179, default JZS BF_+0_ = 0.126).

ILF MD was not significantly lower for *APOE* ε4 carriers than non-carriers (*t*(81.941) = 0.54, *p* = .705, Cohen's *d*_*s*_ = 0.101). BFs again provided evidence in support of the null (default JZS BF_+0_ = 0.142, replication BF_10_ = 0.446). Removing males had no notable impact on the results (*t*(55.856) = 0.818, *p* = .792, Cohen's *d*_*s*_ = 0.187, default JZS BF_+0_ = 0.144, replication BF_10_ = 0.613) nor did removing *APOE* ε2 carriers (*t*(75.242) = 0.713, *p* = .761, Cohen's *d*_*s*_ = 0.145, default JZS BF_+0_ = 0.137).

#### 3.1.3 TBSS

Consistent with the tractography analysis, PHCB-restricted TBSS analysis revealed no significant differences between *APOE* ε4 carriers and non-carriers. This was true of both FA (contrast: carriers > non-carriers) and MD (contrast: carriers < non-carriers). Adopting an uncorrected α level of *p* = .005, as has been done previously (Hodgetts et al., 2019; Postans et al., 2014), did not alter this outcome. Exploratory whole-brain TBSS analysis provided complementary evidence, with no differences evident between *APOE* ε4 carriers and non-carriers.

### 3.2 Secondary (extension) analyses

**Fig. 3:**
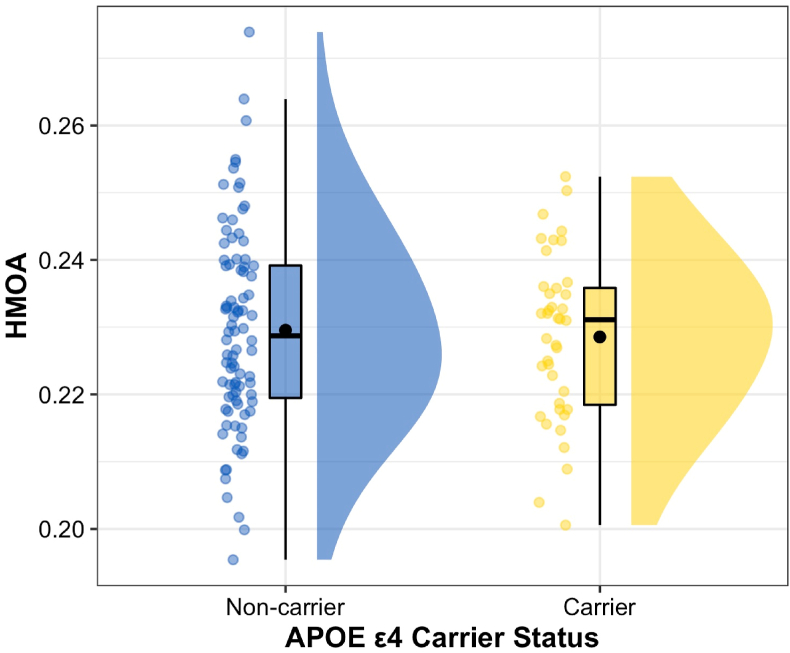
*Differences in PHCB HMOA Between APOE ε4 Carriers and Non-Carriers* *Note*. Differences in HMOA (range = 0–1) between *APOE* ε4 carriers and non-carriers are shown. Individual data points, each representing a single participant, are shown alongside boxplots and density plots (“raincloud plots”; Allen et al., 2021). A small amount of jitter has been added to each data point for clarity. To facilitate interpretation, the mean value (black circle) and median value (a black line) for each group are both shown. Abbreviations: HMOA = hindrance modulated orientational anisotropy.

HMOA values for the PHCB – separated by *APOE* ε4 carrier status – are shown in Fig. 3. Analysis revealed no significant difference between *APOE* ε4 carriers and non-carriers in terms of PHCB HMOA (*t*(90.357) = −0.399, *p* = .691, Cohen's *d*_*s*_ = −0.073). BF analysis also provided moderate evidence in favour of the null (default JZS BF_10_ = 0.215). These results were largely unaffected by the removal of males (*t*(58.33) = 0.445, *p* = .658, Cohen's *d*_*s*_ = 0.10, default JZS BF_10_ = 0.258) or the removal of ε2 carriers (*t*(85.926) = −0.844, *p* = .401, Cohen's *d*_*s*_ = −0.167, default JZS BF_10_ = 0.283).

For completeness, the same analysis was conducted for ILF HMOA. Results revealed that *APOE* ε4 carriers and non-carriers did not differ significantly in terms of ILF HMOA (*t*(94.682) = −0.762, *p* = .448, Cohen's *d*_*s*_ = −0.139). BF analysis provided complementary evidence, largely favouring the null (default JZS BF_10_ = 0.251). This remained the case when males were removed (*t*(48.941) = 0.394, *p* = .696, Cohen's *d*_*s*_ = 0.092, default JZS BF_10_ = 0.256) and when individuals possessing the ε2 allele were removed (*t*(84.914) = −0.819, *p* = .415, Cohen's *d*_*s*_ = −0.162, default JZS BF_10_ = 0.279).

## 4 Discussion

In this study, we aimed to replicate Hodgetts et al.’s (2019) finding that healthy young *APOE* ε4 carriers show higher FA and lower MD than non-carriers in the PHCB but not the ILF. Such a pattern would be in line with suggestions that individuals with pre-existing “hyper-connectivity” between posteromedial cortex and the medial temporal lobe may be more vulnerable to amyloid-β accumulation (Buckner et al., 2009; Bero et al., 2012; de Haan et al., 2012; Jagust and Mormino, 2011; Myers et al., 2014) and/or tau spread (Jacobs et al., 2018; Ziontz et al., 2021) in later life. Extending this work, we also conducted analyses on HMOA, a measure that is proposed to be more sensitive to alterations in tract microstructure than measures derived from the diffusion tensor model, such as FA or MD (Dell’Acqua et al., 2013).

In contrast to the original study, we did not observe higher FA or lower MD in the PHCB of young *APOE* ε4 carriers compared to non-carriers. Rather, for the PHCB, we found: no statistically significant effects in the expected direction (all *p*s ≥ .482); relatively small effect sizes (Cohen's *d*_*s*_ range from −0.183 to 0.341); and BFs providing evidence in favour of the null (default JZS BF_+0_ range from 0.092 to 0.246, replication BF_10_ range from 0.054 to 0.273). Crucially, these BFs represent moderate-to-strong evidence in support of the null hypothesis (Lee and Wagenmakers, 2013). As such, we not only failed to replicate the effect reported by Hodgetts et al. (2019), but also found evidence against the presence of such an effect. Null results were also observed for PHCB HMOA, suggesting that the failure to replicate the original finding cannot readily be attributed to subtle effects that may, or may not, be missed by measures derived from the diffusion tensor model. Nevertheless, there are several plausible explanations for the discrepancy between studies, although they are not necessarily mutually exclusive.

First, it could be the case that Hodgetts et al.’s (2019) findings were false positives. Hodgetts et al.‘s study included just 15 participants in the *APOE* ε4 carrier and non-carrier groups and, as such, was likely underpowered to detect an effect of the magnitude one might expect from this common genetic variant, especially in early adulthood (Dell’Acqua et al., 2015; Henson et al., 2020; Mentink et al., 2021). Given that low statistical power reduces the probability that an observed effect represents a true effect (Button et al., 2013), it is possible that the effects reported by Hodgetts et al. were false positives. However, it is unclear how this relates to evidence of increased posteromedial activity and intrinsic functional connectivity in young adult *APOE* ε4 carriers (Filippini et al., 2009; although see Mentink et al., 2021), including Hodgetts et al.‘s observation that PHCB microstructure correlated with increased posteromedial cortex activity during perceptual scene discrimination (see also Shine et al., 2015). It is also unclear how this relates to prior research reporting that *APOE* ε4 is associated with resting-state oscillatory hyperconnectivity in posteromedial cortex (assessed via magnetoencephalography) among a subset of the current sample (Koelewijn et al., 2019). Regardless, the BF analyses conducted here did provide complementary support for this assertion that the original effects were false positives, demonstrating that the observed data favoured the null. Taken at face value, this interpretation casts some doubt on the notion that *APOE* ε4-related increases in structural connectivity between posteromedial cortex and the medial temporal lobe – as indexed by individual differences in PHCB microstructure – may enhance vulnerability to amyloid-β accumulation and/or tau spread.

Alternatively, it could be the case that Hodgetts et al. (2019) observed a true effect, but its magnitude was exaggerated. Effect size inflation is most likely to occur in studies with small sample sizes, a phenomenon referred to as the “winner's curse” (Button et al., 2013). If true, the analysis reported in this replication attempt might itself be underpowered to detect the effect of *APOE* ε4 on PHCB microstructure (FA, MD, HMOA), thereby constituting a series of Type II errors or false negatives. Such an explanation would help to reconcile the observed findings with prior results indicating that *APOE* ε4 does have an impact on posteromedial structural connectivity early in life (Brown et al., 2011; Felsky and Voineskos, 2013; Hodgetts et al., 2019). While this cannot currently be ruled out, it should be noted that an effect size sensitivity analysis revealed that the smallest effect size detectable at 80% power in the current study was Cohen's *d*_*s*_ = 0.57. In addition, the BF analyses conducted here indicated that the observed data provided moderate-to-strong evidence in favour of the null, as opposed to simply providing inconclusive evidence. This shows that, even with the current sample size, our findings have relatively high evidential value (Dienes, 2014).

Discrepancies in sample characteristics between the current study and Hodgetts et al.’s (2019) study might also offer an explanation for the failure to replicate. For example, as highlighted in Table 1, Hodgetts et al.‘s study included participants that were somewhat younger on average than those included here. Notably, a recent large-scale study found marked age-related variations in white matter microstructure in university students aged 18–26, particular in the cingulum bundle (Tsuchida et al., 2021). Consequently, we cannot rule out the possibility that the effect of *APOE* ε4 on PHCB microstructure is restricted to a specific period of development, such as adolescence or very early adulthood (although see Dell’Acqua et al., 2015). Indeed, Hodgetts et al. themselves originally speculated that *APOE* ε4 carriers and non-carriers undergo different rates of white matter maturation, perhaps via reduced or delayed axonal pruning (Chung et al., 2016), leading to an initial “overshoot” in PHCB microstructure (higher FA, lower MD) among *APOE* ε4 carriers. While longitudinal research is needed to assess this claim, it would help explain why the current study failed to observe the original effect (although see Supplementary Analysis 1). Relatedly, it is possible that the *APOE* ε4 carrier and non-carrier groups included in the two studies differed in known but unobserved moderators of the allele's effect. Relevant factors include obesity (Mole et al., 2020), physical activity (Pearce et al., 2022), diet (Yassine and Finch, 2020), and bilingualism (Vila-Castelar et al., 2022), none of which were reported here or by Hodgetts et al. This might at least partly explain why we failed to replicate the effect originally reported by Hodgetts et al., although future well-powered prospective studies are needed to evaluate the extent to which such factors moderate *APOE* ε4's purported effect on brain structure early in life.

## 5 Summary

In this study, we failed to replicate Hodgetts et al.’s (2019) finding that, relative to non-carriers, healthy young adult *APOE* ε4 carriers show higher FA and lower MD in the PHCB but not the ILF. Rather, the observed data strongly supported the null hypothesis of no difference. The inclusion of a more advanced measure of microstructure – HMOA – did not reveal any further *APOE* ε4-related differences. Our findings thus suggest that young adult *APOE* ε4 carriers and non-carriers do not show differences in PHCB microstructure, casting some doubt on the notion that variation in the microstructural properties of this tract might enhance vulnerability – via excessive connectivity-dependent neural activity – to amyloid-β accumulation and/or tau spread. While these findings do not rule out the possibility that other aspects of posteromedial structural/functional connectivity, neural activity, and/or metabolism may be altered in young adults *APOE* ε4 carriers, they add to a growing number of null findings in this field of research (Costigan et al., 2021; Mentink et al., 2021).

## Funding

This work was supported by a departmental PhD studentship from the 10.13039/501100008533School of Psychology, Cardiff University to R.L., and a 10.13039/100004440Wellcome Strategic Award (104943/Z/14/Z) to C.J.H and K.S.G. Testing of the cohort was supported by the National Centre for Mental Health, supported by funds from 10.13039/100012068Health and Care Research Wales (formerly 10.13039/100009250National Institute for Social Care and Health Research) (Grant No. BR09).

## CRediT authorship contribution statement

**Rikki Lissaman:** Conceptualization, Formal analysis, Visualization, Writing – original draft, Writing – review & editing. **Thomas M. Lancaster:** Resources, Writing – review & editing. **Greg D. Parker:** Methodology, Software. **Kim S. Graham:** Supervision, Writing – review & editing. **Andrew D. Lawrence:** Conceptualization, Supervision, Writing – review & editing. **Carl J. Hodgetts:** Conceptualization, Supervision, Writing – review & editing.

## Declaration of competing interest

The authors declare no competing financial or non-financial interests.
