# Supplementary material for: Tract-specific differences in white matter microstructure between young adult APOE ε4 carriers and non-carriers: A replication and extension study
Source: Neuroimage Rep. 2022 Sep 6;2(4):100126. doi: 10.1016/j.ynirp.2022.100126 (PMC9726682; doi:10.1016/j.ynirp.2022.100126)
Supplement: Multimedia component 1 [file mmc1.docx]

**Supplementary material for ‘Tract-specific differences in white matter microstructure between young adult *APOE* ε4 carriers and non-carriers: A replication and extension study’**

Rikki Lissaman, Thomas M. Lancaster, Greg D. Parker, Kim S. Graham, Andrew D. Lawrence, & Carl J. Hodgetts

| **Supplementary Table 1**  *Comparison of the MRI Scan Parameters Used in the Current Study and in Hodgetts et al. (2019)* | | | | | |
| --- | --- | --- | --- | --- | --- |
|  | | Current Study | | Hodgetts et al. (2019) | |
| *Diffusion sequence* | |  | |  | |
| TE | | 89ms | | 87ms | |
| Voxel dimensions | | 2.4 x 2.4 x 2.4mm | | 2.4 x 2.4 x 2.4mm | |
| FOV | | 230 x 230mm | | 230 x 230mm | |
| Acquisition matrix | | 96 x 96 | | 96 x 96 | |
| Slices | | 60 (aligned AC/PC) with 2.4mm thickness, no gap | | 60 slices (oblique axial) with 2.4mm thickness, no gap | |
| Diffusion gradients | | 30 isotropic directions (b = 1200 s/mm^2^), 3 non-diffusion images (b = 0 s/mm^2^) | | 30 isotropic directions (b = 1200 s/mm^2^), 3 non-diffusion images (b = 0 s/mm^2^) | |
| *Structural sequence* | |  | |  | |
| TR | | 7.8s | | 7.8s | |
| TE | | 3s | | 3s | |
| Voxel dimensions | | 1 x 1 x 1mm | | 1 x 1 x 1mm | |
| FOV | | 256 x 256 x 168mm - 256 x 256 x 180mm | | 256 x 256 x 176mm | |
| Acquisition matrix | | 256 x 256 x 168 – 256 x 256 x 180 | | 256 x 256 x 176 | |
| Flip angle | | 20° | | 20° | |

| *Note*. The diffusion MRI and structural MRI sequences are remarkably similar across studies. In addition, the sequences were run on the same GE SIGNA HDx 3T MRI system, thereby reducing the likelihood that differences in data acquisition can readily account for any discrepancies in results. Abbreviations: AC/PC = anterior commissure/posterior commissure, FOV = field of view, TE = echo time, TR = repetition time. |
| --- |

**Supplementary Figure 1**


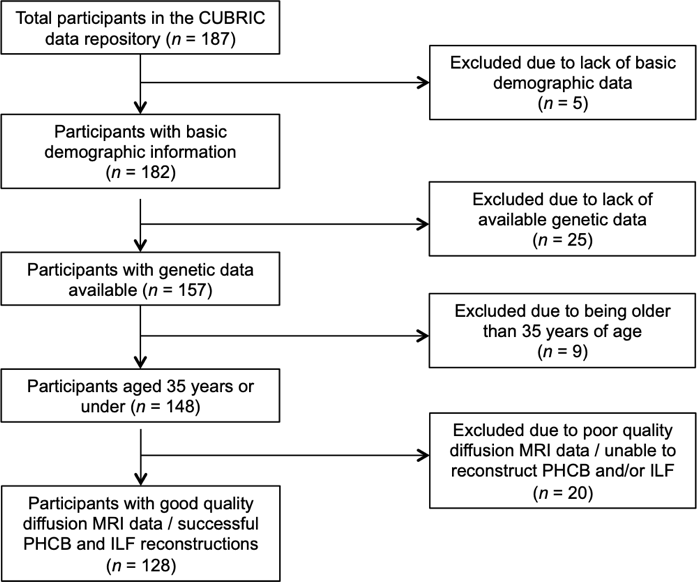
*Flowchart of the Progress from Data Availability to Inclusion in the Study*

*Note.* Of the total number of participants in the CUBRIC data repository, approximately two-thirds (68.45%) were included in the final sample reported here. All exclusions are reported in the main body of the manuscript. Abbreviations: CUBRIC = Cardiff University Brain Research Imaging Centre, ILF = inferior longitudinal fasciculus, PHCB = parahippocampal cingulum bundle.

**Supplementary Analysis 1**

To examine whether there were subsets of participants in our sample who showed the original effect reported In Hodgetts et al. (2019), we conducted a bootstrap-style analysis:

*APOE* ε4+

(*n* = 40)

*APOE* ε4-

(*n* = 88)

**Randomly select participants with *n* per group matching original study**

*APOE* ε4+

(*n* = 15)

*APOE* ε4-

(*n* = 15)

Calculate Cohen’s *d* for difference in FA / MD

**Repeat 1000 times with replacement**

The observed effect size distributions for FA and MD are shown below. For FA, the mean Cohen’s *d* value from the randomly generated samples was -0.104. For MD, the mean Cohen’s *d* value from the randomly generated samples was 0.277.

Only 12 randomly generated samples produced effect sizes of the size and direction (Cohen’s *d* >= 0.62) reported by Hodgetts et al. (2019) for FA. This represents 1.2% of all randomly generated samples. No randomly generated samples produced effect sizes of the size and direction (Cohen’s *d* <= -0.84) reported by Hodgetts et al. for MD.

The two tables below show the basic sample characteristics (age, sex) for the randomly generated samples that produced Cohen’s *d* values equal to or greater than that reported by Hodgetts et al. (2019) for group differences in FA. As shown, these participants appear relatively similar to those in the full cohort.

**FA**


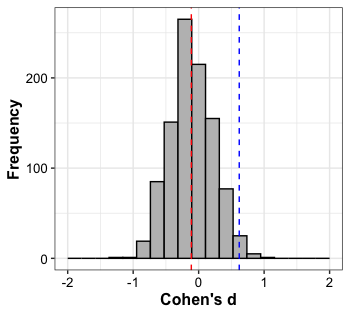


*Note*. The Cohen’s *d* value observed in the current study (*d* = -0.112) is highlighted by the dashed red line, whereas the Cohen’s *d* value observed by Hodgetts et al. (2019) (*d* = 0.62) is highlighted by the dash blue line.

**MD**


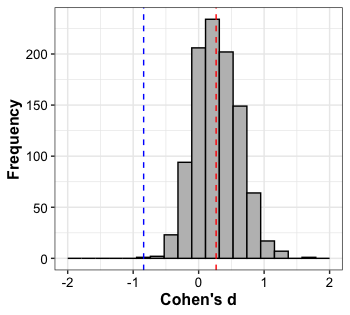


*Note*. The Cohen’s *d* value observed for MD in the current study (*d* = 0.267) is highlighted by the dashed red line, whereas the Cohen’s *d* value observed for MD by Hodgetts et al. (2019) (*d* = -0.84) is highlighted by the dash blue line.

| Mean age of randomly generated *APOE* ε4 carrier (*n* = 15) and non-carrier (*n* = 15) groups that produced Cohen’s d values for FA equivalent to, or larger than, that reported by Hodgetts et al. (2019) | | | | | | | | | | | | | |
| --- | --- | --- | --- | --- | --- | --- | --- | --- | --- | --- | --- | --- | --- |
|  | **1** | **2** | **3** | **4** | **5** | **6** | **7** | **8** | **9** | **10** | **11** | **12** | **Grand Mean** |
| ***APOE* ε4+** | 24.7 | 24.7 | 24.6 | 23.5 | 25.6 | 23.7 | 23.6 | 24.9 | 23.1 | 23.4 | 24.3 | 23.1 | 24.1 |
| ***APOE* ε4-** | 23.1 | 23.8 | 22.5 | 25.1 | 23.4 | 23.4 | 24.1 | 22.8 | 23.9 | 23.7 | 23.4 | 23.3 | 23.54 |
| *Note.* The grand mean values do not differ substantially vs. those reported in the full sample (*APOE* ε4+ = 23.9, *APOE* ε4- = 23.7). Abbreviations: *APOE* ε4+ = *APOE* ε4 carrier, *APOE* ε4- = *APOE* ε4 non-carrier. | | | | | | | | | | | | | |

| Distribution of males and females among randomly generated *APOE* ε4 carrier (*n* = 15) and non-carrier (*n* = 15) groups that produced Cohen’s d values for FA equivalent to, or larger than, that reported by Hodgetts et al. (2019) | | | | | | | | | | | | | | | |
| --- | --- | --- | --- | --- | --- | --- | --- | --- | --- | --- | --- | --- | --- | --- | --- |
|  |  | **1** | **2** | **3** | **4** | **5** | **6** | **7** | **8** | **9** | **10** | **11** | **12** | **Median** | **%** |
| ***APOE* ε4+** | **Female** | 9 | 10 | 9 | 12 | 9 | 10 | 10 | 8 | 11 | 10 | 9 | 11 | 10 | 66.66 |
|  | **Male** | 6 | 5 | 6 | 3 | 6 | 5 | 5 | 7 | 4 | 5 | 6 | 4 | 5 | 33.33 |
| ***APOE* ε4-** | **Female** | 11 | 10 | 10 | 11 | 10 | 12 | 11 | 11 | 9 | 7 | 10 | 10 | 10 | 66.66 |
|  | **Male** | 4 | 5 | 5 | 4 | 5 | 3 | 4 | 4 | 6 | 8 | 5 | 5 | 5 | 33.33 |
| *Note.* The distribution of males and females per group does not differ substantially vs. those reported in the full sample (*APOE* ε4+ females = 28 [70%], *APOE* ε4+ males = 12 [30%], *APOE* ε4- females = 58 [66%], *APOE* ε4- males = 30 [34%]). Abbreviations: *APOE* ε4+ = *APOE* ε4 carrier, *APOE* ε4- = *APOE* ε4 non-carrier. | | | | | | | | | | | | | | | |
